# Development of Multi-Target Chemometric Models for the Inhibition of Class I PI3K Enzyme Isoforms: A Case Study Using QSAR-Co Tool

**DOI:** 10.3390/ijms20174191

**Published:** 2019-08-27

**Authors:** Amit Kumar Halder, M. Natália Dias Soeiro Cordeiro

**Affiliations:** Department of Chemistry and Biochemistry, University of Porto, 4169-007 Porto, Portugal

**Keywords:** PI3K inhibitors, cancer, QSAR, multi-target models, linear discriminant analysis, random forest

## Abstract

The present work aims at establishing multi-target chemometric models using the recently launched quantitative structure–activity relationship (QSAR)-Co tool for predicting the activity of inhibitor compounds against different isoforms of phosphoinositide 3-kinase (PI3K) under various experimental conditions. The inhibitors of class I phosphoinositide 3-kinase (PI3K) isoforms have emerged as potential therapeutic agents for the treatment of various disorders, especially cancer. The cell-based enzyme inhibition assay results of PI3K inhibitors were curated from the CHEMBL database. Factors such as the nature and mutation of cell lines that may significantly alter the assay outcomes were considered as important experimental elements for mt-QSAR model development. The models, in turn, were developed using two machine learning techniques as implemented in QSAR-Co: linear discriminant analysis (LDA) and random forest (RF). Both techniques led to models with high accuracy (ca. 90%). Several molecular fragments were extracted from the current dataset, and their quantitative contributions to the inhibitory activity against all the proteins and experimental conditions under study were calculated. This case study also demonstrates the utility of QSAR-Co tool in solving multi-factorial and complex chemometric problems. Additionally, the combination of different in silico methods employed in this work can serve as a valuable guideline to speed up early discovery of PI3K inhibitors.

## 1. Introduction 

Machine learning-based chemometric modelling is widely applied to the design and discovery of new therapeutic agents with superior biological activities. It is now being realised that ligand–protein interaction-based traditional drug discovery methods may no longer be sufficient to satisfy clinical drug safety criteria. ‘Data fusion’ techniques that incorporate multiple structural, genetic and pharmacological data types and sources are now becoming essential for the discovery of safe and effective therapeutic agents [1,2]. Quantitative structure–activity relationship (QSAR), which relates the variations in the observed activity to numerical descriptors, is extensively used to find structural requirements for higher active molecules and/or to predict activity of novel compounds [3,4,5]. Conventional QSAR models are developed based on the experimental activity of the compounds tested against a single biological target following a specific experimental condition. However, multi-target QSAR models have drawn considerable attention very recently. It is worth mentioning also that the ‘multi-target’ term is used here to define those chemometric models that truly integrate the data for simultaneous prediction of response parameters against multiple biological targets under different experimental conditions [6,7,8,9,10]. Our group has launched QSAR-Co [11], a publicly available Java-based tool to support Box–Jenkins based mt-QSAR model development, validation and screening (https://sites.google.com/view/qsar-co). In order to understand how this tool may perform with diverse and complex datasets containing multi-factorial response variables, we performed a case study with this QSAR-Co tool considering the inhibitors of different class I phosphoinositide 3-kinases (PI3Ks) isoforms as potential anticancer agents. As per our literature search, the current case study is the first report on the multi-target chemometric modelling on the inhibition of class I PI3K enzyme isoforms.

The phosphoinositide 3-kinases (PI3Ks) belong to a large lipid enzyme family involved in the phosphorylation of the 3’-OH group of phosphatidylinositols (PI) present in the plasma membranes [12]. In response to various external stimuli such as growth factors, hormones and environmental variations, PI3Ks generate intracellular signals to regulate diverse cellular processes such as cell proliferation, survival, differentiation, migration, inflammation and metabolism [13]. Based on their structural- and enzymatic-kinetic differences, PI3Ks are classified into three classes, i.e., class I, class II and class III [14]. Among all these, only class I PI3Ks are able to phosphorylate membrane substrate phosphorylate phosphatidylinositol-4,5-bisphosphate (PIP_2_) to produce a second messenger named phosphatidylinositol-3,4,5-trisphosphate (PIP_3_), which subsequently initiates a signalling cascade for activation of the downstream effectors to trigger increased cell growth, metabolism and cell-cycle progression [15,16]. The class I PI3Ks are further categorised into class IA and IB enzymes. The class IA includes three enzyme isoforms, namely: PI3Kα, PI3Kβ and PI3Kδ; each isoform consists of a p110 catalytic unit (p110α, p110β and p110δ) that forms heterodimers with a regulatory subunit. On the other hand, the class IB includes only one enzyme isoform PI3Kγ in which the heterodimer is formed between catalytic subunit p110γ and a regulatory subunit [12,17]. Each PI3K isoform is found to possess distinct tissue specificity as well as unique physiological functionalities. The PI3Kα and PI3Kβ are expressed ubiquitously whereas the expressions of PI3Kδ and PI3Kγ are limited only to hematopoietic system [18,19]. PI3Kα is involved in regulating glucose homeostasis, insulin signalling and mitochondrial growth. PI3Kβ promotes platelet adhesion and aggregation, playing significant roles in the progression of thrombotic diseases [15,20]. Additionally, PI3Kβ participates in several immune responses. Both PI3Kγ and PI3Kδ play significant roles in inflammation and immunisation [21,22]. Due to their crucial involvement in various physiological functions, it is not surprising that class I PI3K inhibitors are implicated in the treatment of a range of diseases such as arthritis [22], thrombosis, asthma [23], inflammation, cardiovascular diseases, PIK3CA-related overgrowth syndromes, etc. Nevertheless, class I PI3K inhibitors are above all well-recognised as potential anticancer agents. Dysregulation of the PI3K pathway leads to severe abnormality in the cell cycle, cell growth survival, metabolism and motility, which are some common hallmarks of cancer [17,18,24]. A number of PI3K inhibitors, including pan-PI3K inhibitors, isoform-selective PI3K inhibitors and dual PI3K/mTOR inhibitors are currently under clinical trials for cancer treatment [24]. Idelalisib, a PI3Kδ-specific inhibitor, has been approved by the United States Food and Drug Administration (USFDA) for the treatment of follicular non-Hodgkin’s B-cell lymphoma, small-cell lymphocytic lymphoma and chronic lymphoid leukemia [25]. Two other PI3K inhibitors, that is duvelisib (PI3Kγ/δ inhibitor) and copanlisib (pan-PI3K inhibitor), have also found approval for the treatment of leukemia and lymphoma [26]. The p110α encoding gene *PIK3CA* is one of the most frequently mutated genes in most human cancers, especially breast, ovarian and colorectal cancers [17,18,27]. Although p110β catalytic subunit mutations are not commonly found, its tumorigenic potential is associated with the loss of the enzyme phosphatase and tensin homolog (known as PTEN), a negative regulator of PI3K pathway that dephosphorylates PIP3 [24,28]. While PI3Kδ signalling is associated with the activation, proliferation and survival of the B cells [29], PI3Kγ is activated in response to tissue hypoxia and plays crucial roles in the development of tumour microenvironment [30]. Due to their immunomodulatory potencies, PI3Kδ and PI3Kγ inhibitors are potential candidates for cancer immunotherapy [29]. Nevertheless, inhibition of PI3Kδ and PI3Kγ may give rise to moderate to severe adverse immune responses. It is worth mentioning here that despite promising therapeutic results, similar to many targeted cancer therapies, different types of PI3K inhibitors (pan-/isoform-specific) may give rise to serious adverse effects that restrict their clinical applications [31]. Very recently, Curigliano and Shah reported a detailed review of the adverse effects obtained from different clinically tested class I PI3K inhibitors [29]. It is generally postulated that pan-PI3K inhibitors may exhibit higher efficacy against cancer cells whereas isoform-specific PI3K inhibitors may have less overall toxicity [15,31]. While overall efficacy and relative benefit of pan- and isoform-specific PI3K inhibitors will be decided through clinical trials, a detailed understanding of the structural and physicochemical factors responsible for higher potency and selectivity towards different class I PI3K inhibitors will definitely help in the development of the next generation of PI3K inhibitors. 

So far, a number of different ligand-based in silico methods and protocols have been employed by different researchers for the discovery of PI3K inhibitors, and these include 2D-quantitative structure–activity relationship (2D-QSAR) modelling, 3D-QSAR, 3D-pharmacophore mapping, etc. [32,33,34,35,36,37,38,39,40,41,42,43,44,45,46,47,48,49]. However, all these methods suffer from at least one of the following shortcomings. From one side, only a limited number of data samples have been considered for modelling and therefore, the overall applicability of these models may be limited. On the other hand, only one PI3K enzyme isoform has been considered as the biological target for modelling. Considering the multi-factorial nature of the diseases, these single target models may also pose serious limitations to the applicability of these in silico models. Herein we report, for the first time, a multi-target in silico chemometric modelling with different isoforms of class I PI3K inhibitors. Even though Liew et al. earlier performed a consensus QSAR modelling with an integrated dataset containing inhibition data for all four class I PI3K inhibitors, the final models could not differentiate among inhibitory activities against different PI3K isoforms [36]. The mt-QSAR models developed for the current case study, apart from being highly predictive in nature, are also capable of differentiating among different biological targets as well as experimental assay conditions. Based on the mt-QSAR model, contributions of some structural fragments for higher/lower inhibitory potency against PI3K enzymes are also discussed. 

## 2. Results and Discussion

### 2.1. Linear Mt-QSAR Model Development 

The major concepts and design strategies of QSAR-Co have been reported elsewhere [11]. A step-by-step instruction manual for the use of this tool is also available online (https://sites.google.com/view/qsar-co). The multi-target modelling of the QSAR-Co tool is based on the Box–Jenkins moving average approach [10,11,50]. The tool currently allows for the development of a linear mt-QSAR model by genetic algorithm based linear discriminant analysis (GA-LDA) technique [11,51,52,53]. A dataset comprising 726 compounds were collected from CHEMBL (https://www.ebi.ac.uk/chembl/). As can be observed from the dataset (provided in supplementary material, SM1.xlsx), each compound was tested against class I PI3K enzymes by a specific cell-based assay. In cell-based assays, the enzyme inhibitory potency of the inhibitors is measured in a specific cell line, and the outcomes of these assays are thus affected not only by the type of biological target (specific class I PI3K enzyme isoform, in this case) but also by the complex multi-factorial conditions that exist inside a specific cellular system [54]. The rationale and methodology of resorting to the Box–Jenkins moving average approach for setting up the present mt-QSAR modelling have been documented in detail previously [10,11,50], thus an overview highlighting only the most principal technical aspects will be given here. Prior to model development, the experimental elements for each data point are settled by the Box–Jenkins approach [11]. Any experimental element may simply be defined as the specific condition of the assay which is likely to alter the assay results of the compound under study. The first element considered in the current work is the biological enzyme target or *bt*. Each dataset compound was assayed against at least one of the four above-mentioned class I human PI3K enzymes (*bt*). Moreover, 34 different types of cell lines were found in the retrieved dataset (SM1.xlsx). Some of these cell lines are wild types whereas the others are found to have at least one mutation. Furthermore, in some assays PTEN-deficient or PTEN-null human cell lines were used. Therefore, it may be inferred that depending on the cell type, the experimental outcomes should also vary to a considerable extent. Depending on the nature of the cell lines, we considered two more experimental elements, namely *cl* (type of cell line) and *mt* (wild or mutated) for the multi-target modelling. Overall, each combination of these three elements *bt, cl* and *mt* defines a specific experimental condition, which may be expressed as an ontology of the form *cj* → (*bt, cl, mt*). It is worth mentioning here that some compounds of the dataset have been assayed against more than one experimental element. Depending on the biological response, each dataset sample *i* was annotated as active [*IAi*(*cj*) = 1] or inactive [*IAi*(*cj*) = −1]. The response variable *IAi*(*cj*) is a binary variable characterising the inhibitory potency of the *i*th sample under a specific experimental condition *cj*. Any compound with IC_50_/K_i_/K_d_ values ≤ 600 nM was assigned as active whereas the remaining data samples considered as inactive. Generally speaking, any compound exhibiting an inhibitory activity in the micromolar range is considered as a ‘*hit*’ molecule in the context of drug discovery [55]. In this work, the selected cut-off value appears in the sub-micromolar range to make the models more rigorous for the selection of more potent inhibitors. This cut-off also prevented excessive imbalance between active and inactive data points. 

Details about data curation, dataset division, molecular descriptor calculation, descriptor modification, data-pre-treatment and model development are described in the Materials and Methods section. In the Box–Jenkins approach, each molecular descriptor calculated for a dataset compound is modified through a systemic procedure so that contributions of each experimental element may be easily incorporated (see Section 3.2). The mt-QSAR model was established with a modelling dataset, which is a combination of a sub-training set (*n* = 453) on which the model was developed, and a test set (*n* = 113), which was used to validate the model.

The resulting best-fit mt-QSAR-LDA model found (a ten-variable equation) is given below along with the statistical parameters of the LDA.

(1) $$\begin{array}{l} IAi(cj)=\;+1.070\;+\;16.121\;\Delta [\mathrm{SpMAD}\_A]b_{t}\;+\;5.979\;\Delta [\mathrm{HATS}2i]b_{t}\;+\;5.582\;\Delta [\mathrm{nROCON}]c_{l}\\ +\;0.475\;\Delta [F07[N\text{-}N]]{c_{l}}_{\;}+\;0.131\;\Delta [\mathrm{HTm}]m_{t}\;+\;0.069\;\Delta [\mathrm{SM}15\_\mathrm{EA}(\mathrm{dm})]c_{l}\;-\;2.898\;\Delta [\mathrm{nCONN}]b_{t}\\ -2.662\;\Delta [R1m]c_{l}\;-\;0.905\;\Delta [\mathrm{Mor}18m]b_{t}\;-\;0.850\;\Delta [\mathrm{HATS}8s]c_{l} \end{array}\;N=\;453,\;\lambda \;=\;0.234,\;\chi^{2}\;=\;648.27,\;D^{2}\;=\;11.522,\;F\;(10,442)\;=\;144.89,\;p\text{-}\mathrm{value}\;<\;{10^{-}}^{16}$$

As can be seen, this mt-QSAR-LDA model is satisfactory in both statistical significance, goodness of fit and robustness. The low value found for the Wilks λ statistic (0.234) [56] shows also that the model displays an adequate discriminatory power. The classification results obtained for the sub-training and test sets are presented in Table 1, outlining the overall performance of the present mt-QSAR LDA model.

As can be observed from Table 1, the QSAR model achieved an accuracy of 95.36% and 93.80% for the sub-training and the test sets, respectively. Furthermore, the model correctly classified 95.68% of the active samples and 94.57% the inactive ones of the sub-training set. Similarly, the model could correctly classify 94.38% of active and 91.67% of inactive samples of the test set, respectively. These findings corroborate that this mt-QSAR-LDA model is highly capable of discriminating active PI3K inhibitors from inactive ones. The high values found for the Matthews correlation coefficients (i.e., 0.889 for the sub-training and 0.825 for the test set) further confirms the statistical robustness of the model [57].

**Figure 1:**
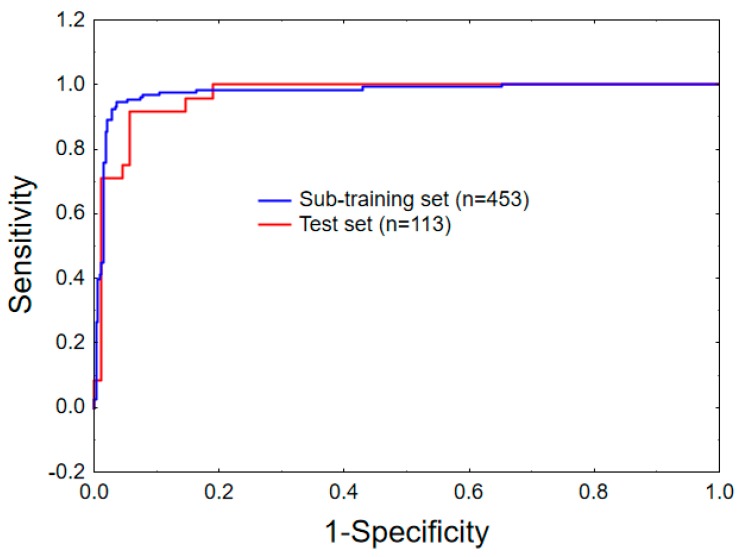
Receiver operating characteristic (ROC) curves for the sub-training (10-fold) and the test sets.

Figure 1 shows the receiver operating characteristic (ROC) plots for the sub-training (10-fold) and the test sets. The area under the ROC curve (AUROC) is another important well-known statistical parameter to evaluate the statistical significance of a classifier model. For a random classifier, an area under ROC (AUROC) value of 0.5 is obtained [58]. In the current case, AUROC values of 0.977 and 0.968 are obtained for the sub-training and the test sets. Therefore, these values further emphasised the high statistical significance of the developed mt-QSAR model. 

Further analysis of this classification model should only be carried out after checking the degree of collinearity among the independent variables of the model, and that may be easily diagnosed by analysing the cross-correlation matrix. As can be seen in Table 2, the maximum Pearson correlation coefficient (i.e., *r*) obtained is 0.74, leading us to infer that the developed model is non-redundant in nature.

Finally, the applicability domain (AD) of the model was determined by the standardisation approach proposed by Roy et al [61] with the help of QSAR-Co [11]. Fifteen data-samples of the sub-training and two samples of the test set were found to remain outside the AD of the model (SM1.xlsx). Overall, it may thus be concluded that the developed mt-QSAR-LDA model satisfies each required criterion for being a robust classifier model.

In addition to model development, the QSAR-Co tool also allows the screening of large datasets. Using this screening facility, the mt-QSAR-LDA model was used to screen an external validation set (*n* = 160) to further confirm the external predictivity. Details of this external dataset and calculated descriptors, as well as the results of the predictions are provided in supplementary materials (SM2.xlsx). The GA-LDA model could correctly predict 118 out of 123 active datapoints achieving a sensitivity of 95.93%. On the other hand, a specificity of 89.19% was obtained as 33 out of 37 inactive data points were predicted correctly. Therefore, as far as the screening of external validation set is concerned, the model has an accuracy of 94.38% and a Matthews correlation coefficient (MCC) value of 0.843. Moreover, when the AD was calculated, only five samples of the external validation set were found as outliers. Altogether, these diverse statistics demonstrate the high internal quality as well as predictive power of the derived mt-QSAR-LDA model.

### 2.2. Physicochemical and Structural Interpretation of the Molecular Descriptors

**Figure 2:**
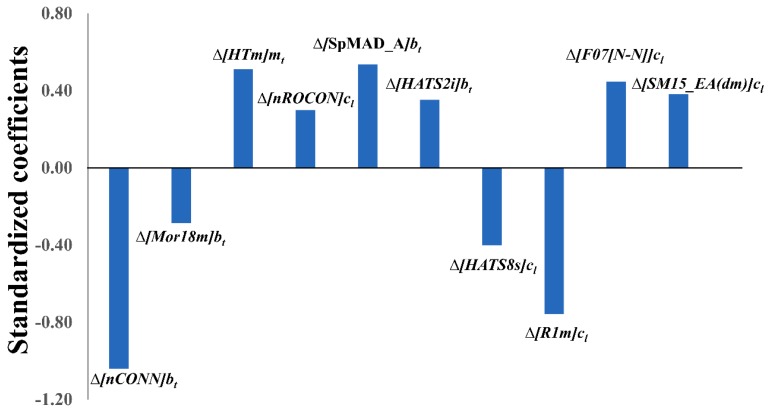
Standardised coefficients *vs.* variables in the mt-QSAR LDA model.

Let us now scrutinise the physicochemical/structural information of the ten variables appearing in the derived mt-QSAR-LDA model (see Equation (1)) using the QSAR-Co software [11]. Evidently, each model variable not only expresses the contribution of a specific molecular descriptor (i.e., core descriptor) but also highlights the significance of a specific experiment element (i.e., *cj*). Therefore, in order to understand the significance of these variables, both these factors (i.e., core descriptor and experimental element) should be taken into consideration. As it is evident, all three experimental elements considered in the current study (i.e., *b_t_, c_l_* and *m_t_*) found a place in the final mt-QSAR-LDA model (Equation (1)). The *c_l_* element, which incorporates the information due to cell-type changes, appeared five times in the model whereas the element representing the biological enzyme target (i.e, *b_t_*) was coupled to four independent variables of the model, meanwhile the third element *mt,* representing the mutation status of the cell, appeared only once. The meaning of these variables is described in Table 3. To understand the relative importance of these model variables, one needs to inspect the absolute values of their standardised coefficients (see Figure 2), that is, those are as follows: Δ[*nCONN*]*b_t_* > Δ[*R1m*]*c_l_* > Δ[*SpMAD_A*]*b_t_* > Δ[*HTm*]*m_t_* > Δ[*F07*[*N-N*]]*c_l_* > Δ[*HATS8s*]*c_l_* > Δ[*SM15_EA*(*dm*)]*c_l_* > Δ[*HATS2i*]*b_t_* > Δ[*nROCON*]*c_l_* > Δ[*Mor18m*]*b_t_*. 

The most significant variable of the model is Δ[*nCONN*]*b_t_*, where *nCONN* is a functional group count descriptor that annotates the number of urea/thiourea fragments present in the structure of the compound. This variable, which is also sensitive towards the biological enzyme target element (*b_t_*), indicates the significance of urea/thiourea molecular fragment for determining the potency of the PI3K inhibitors. The core descriptor of variable Δ[*R1m*]*c_l_* (i.e., *R1m*) stands for the R-autocorrelation of lag-1, obtained through the leverage/geometry matrix of the corresponding molecular graphs. *R1m* is a 3D GETAWAY (GEometry, Topology and Atom Weights AssemblY) type of descriptor [62,63], which attempts to match molecular geometry with chemical information obtained from atomic masses. Furthermore, Δ[*R1m*]*c_l_* also incorporates the changes due the variation in the cell type in which the assay was performed. Both variables Δ[*nCONN*]*b_t_* and Δ[*R1m*]*c_l_* have negative coefficients, whereas the third most significant descriptor Δ[*SpMAD_A*]*b_t_* has a positive coefficient. Δ[*SpMAD_A*]*b_t_* is based on the 2D matrix-based descriptor *SpMAD_A,* which stands for the spectral mean absolute deviation obtained from the adjacency matrix [64,65]. Δ[*HTm*]*m_t_* is the only variable which is modified on the basis of mutation status of the cell, and the core descriptor *HTm* is a 3D GETAWAY descriptor weighted by atomic mass, similar to the *R1m* described above. However, unlike *R1m*, which is obtained from the leverage/geometry matrix, the calculation *HTm* is based on the molecular influence matrix [62,63]. The Δ[*F07*[*N–N*]]*c_l_* is dependent on the 2D-atom pair descriptor *F07*[*N–N*]*,* which is the frequency of N−N at a topological distance of seven [64]. Presence of this descriptor in the model indicates that the distances between two nitrogen atoms in the compound structure may contribute to its inhibitory activity, depending at the same time on the cell type of the enzyme inhibition assay. Both Δ[*HTm*]*m_t_* and Δ[*F07*[*N–N*]]*c_l_* are positively correlated with the response variable.

The mt-QSAR model showed the importance of two other 3D-GETAWAY based descriptors, which are Δ[*HATS8s*]*c_l_* and Δ[*HATS2i]b_t_* [62,63]. While Δ[*HATS8s*]*c_l_* has a negative coefficient, Δ[*HATS2i*]*b_t_* is found to be associated with a positive one. Significantly, both these descriptors are calculated on the basis of the leverage weighted autocorrelation of molecular graphs. As atomic weights, intrinsic state (*I*-state) and ionization potential are used for the calculation of *HATS8s* and *HATS2i* descriptors, respectively [62,63]. Another descriptor Δ[*SM15_EA*(*dm*)]*c_l_* is based on the 2D-edge adjacency spectral moment descriptor *SM15_EA*(*dm*). Similar to 2D matrix-based adjacency matrix descriptors, edge adjacency matrices are also calculated considering the chemical structures as weighted graphs. In the case of edge adjacency indices however, the elements of *edges* are substituted by the bond orders between connected atoms in the molecule [66]. Like Δ[*nCONN*]*b_t_*, another variable of the model Δ[*nROCON*]*c_l_* is based on a simple functional group count descriptor. The core descriptor of the latter, *nROCON,* annotates the number of aliphatic carbamates or thiocarbamates in the compounds. Unlike Δ[*nCONN*]*b_t_*, Δ[*nROCON*]*c_l_* is however dependent on the cell type element (*c_l_*). Both Δ[*SM15_EA*(*dm*)]*c_l_* and Δ[*nROCON*]*c_l_* are sensitive to changes of cell types and both are associated with positive coefficients. The last variable of the model Δ[*Mor18m*]*b_t_* is based on the 3D-Morse (molecular representation of structures based on electronic diffraction) descriptor *Mor18m*. 3D-Morse are descriptors calculated by summing atomic weights observed from different angular scattering functions (or signals) [67]. For the calculation of *Mor18m*, the mass is used as atomic weight. Δ[*Mor18m*]*b_t_*, which is dependent on the biological enzyme target, is negatively correlated with the response variable.

### 2.3. Non-Linear Mt-QSAR Model Development 

In addition to GA-LDA based linear model development, QSAR-Co allows for the generating of non-linear mt-QSAR models using the random forest (RF) strategy [11,68,69]. It is often observed (but not always) that non-linear models developed with all calculated descriptors produce more predictive models than the linear models developed with a limited number of descriptors [70,71]. Of course, overall interpretability of such non-linear models is inferior to that of linear models [70,71,72,73]. RF is basically an ensemble classification method that makes predictions by averaging over the predictions of multiple independent decision trees. Due to its high accuracy and superiority, RF has received considerable attention in recent years [68,69]. One of the major advantages of RF is that it is less susceptible to produce overfitted models. As such, RF may be preferred over many other non-linear machine learning methods to produce highly accurate mt-QSAR models [74,75,76]. 

In the current case study, we applied the random forest (RF) based classification approach to develop non-linear models [68,69]. All the descriptors calculated by AlvaDesc were applied for the development of RF model with the help of the QSAR-Co tool [11]. The obtained results for the best RF model found are depicted in Table 4. 

The internal predictivity of the RF model was judged by 10-fold cross-validation statistics (as implemented in QSAR-Co tool) and in so doing, an overall accuracy of 94.92% was obtained. On the other hand, the RF model depicted an accuracy of 95.57% for the test set. However, when the developed RF model was used to predict the external validation set to check its true external predictivity, the model could correctly classify 119 out of 123 active and 28 out of 37 inactive data points. Therefore, the RF model reveals a sensitivity, specificity, accuracy and MCC of 96.75%, 75.68%, 91.88% and 0.763, respectively. Overall, the LDA model could correctly classify 689 out of 726 data-points whereas the RF model correctly classified 685 out of 726 data-points. Evidently, in terms of predictive power, the performance of the LDA model is slightly better than that of the RF model. The attribute importance (based on the average impurity decrease per attribute over the trees) are provided in the supplementary materials (SM3.xlsx).

### 2.4. Quantitative Contributions of the Fragments Towards Inhibitory Activity

**Figure 3:**
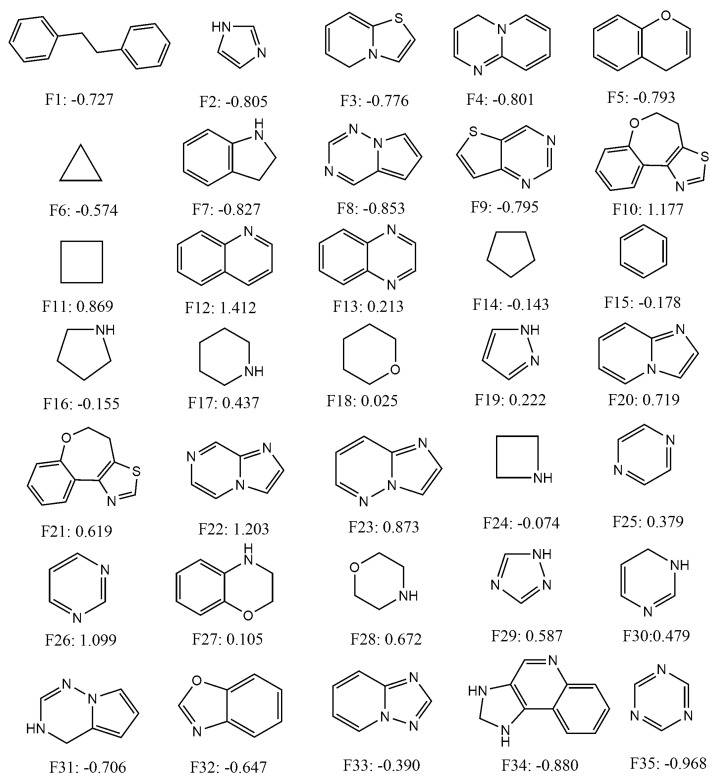
Chemical structures and average standardized scores of selected fragments.

Since the mt-QSAR LDA model was shown to be slightly more predictive than the non-linear mt-QSAR RF model, we attempted to use it as a tool to calculate the quantitative contributions of some important cyclic/ring fragments present in the dataset. To identify such fragments, the Bemis–Murcko scaffolds [77] were calculated with the entire dataset containing 726 data-points with the help of the OCHEM web server [78]. Thirty-five small alicyclic/ring fragments that were found in more than 10 dataset compounds were selected and the inhibitory activities of these fragments were subsequently calculated against all the experimental conditions (i.e., combinations of the elements *b_t_, m_t_* and *c_l_*) reported in this work (a total of 40). After that, the variables appearing in Equation (1) were calculated for these fragments, following the same procedure by which these were calculated for the external validation set molecules, and considering all 40 different experimental conditions. A total of 1400 (= 35 × 40) scores were obtained by putting the calculated variables into Equation (1). These scores are, however, non-standardised and in order to obtain standardised scores the following procedure was adopted. The arithmetic means and the standard deviation of all these non-standardised scores were calculated. Subsequently, a standardisation procedure was employed where the arithmetic scores were subtracted from each non-standardised score and these subtracted values were then divided by the standard deviation [10,50]. These standardised scores represent the quantitative contributions of the fragments for the inhibitory potentials of these fragments and various experimental conditions. The chemical structures and the average standardised scores (or contributions) calculated for each fragment are presented in Figure 3, whereas all these scores are provided as supplementary materials (SM4.xlsx).

As can be deduced from Figure 3, fragments such as **F12, F22, F10, F26, F23, F11, F20, F28, F21** and **F29** depicted highly positive average standardised scores (>0.5) signifying that these fragments had positive contributions against all experimental conditions. These fragments may, therefore, be considered as the most suitable fragments for the design of novel class I PI3K inhibitors. Similarly, some fragments such as **F35, F34, F8, F7, F6, F2, F4, F9, F5, F3, F1, F31** and **F32** displayed highly negative average standardised scores (<−0.5). Thus, the later fragments should be avoided while designing novel PI3K inhibitors. Moderate positive contributions are obtained for **F18, F27, F13, F19, F25, F30** and **F17**, whereas slightly negative contributions are observed for fragments such as **F33, F15, F16, F14** and **F24.** These fragments are likely to have mixed responses against different experimental conditions. It should be noted here that some compounds of the current dataset may be found with positive response even though these contain fragments with negative scores. The opposite case is also true. It is not unnatural because combinations and connections of these fragments ultimately determine the overall inhibitory potential of the compounds, rather than their mere presence in the compound structures. 

## 3. Materials and Methods

### 3.1. Dataset Curation and Descriptor Calculation

After collecting the dataset compounds from CHEMBL (https://www.ebi.ac.uk/chembl/), the dataset was curated by removing duplicate data-points. The SMILES formats of the molecules obtained from the CHEMBL were converted into SDF formats by the MarvinView v18.18.0 software (https://docs.chemaxon.com/display/docs/MarvinView). Molecular descriptors were calculated by the AlvaDesc v.1.0.8 software (https://www.alvascience.com/alvadesc/) with the help of OCHEM [78], a freely available web server for QSAR descriptor calculation, model development and data storage. Before the calculation of these descriptors, the compounds were pre-processed (structures were standardised, neutralised, cleaned and salts were removed) in the OCHEM platform. Furthermore, during descriptor calculation, each dataset compound was geometrically optimised in OCHEM by the Corina software [79].

### 3.2. Box–Jenkins Approach

The global descriptors calculated by AlvaDesc v.1.0.8 only consider the chemical structures of the compounds, and these descriptors are thus incapable of discriminating the influence on the chemical structure when a specific molecule is assayed under more than one experimental condition (i.e., *cj*). To solve this problem, we adopted the Box–Jenkins moving average approach [80], the details of which have been discussed previously in detail [10,11,50,60]. Briefly, Box–Jenkins operators are used to calculate successive average values of a defined property of a defined system at different intervals of time. In Box–Jenkins based mt-QSAR modelling, the time domain is not considered. Rather, the arithmetic average of any molecular descriptors for a specific experimental condition is calculated as follows [11]:

(2) $$avg(D_{i})c_{j}=\sum_{i=1}^{n(c_{j})}D_{i}$$

Here, *D_i_* is the calculated descriptor of the individual compound ‘*i*’ and *n*(*c_j_*) is the number of actives in the modelling dataset (= sub-training set + test set) assayed under the same element of the experimental condition *c_j_*. The *avg*(*D_i_*)*c_j_* is thus the arithmetic mean of the descriptors (*D_i_*) for a specific experimental condition (*c_j_*). After generating the *avg*(*D_i_*)*c_j_* values, the final modified descriptors (Δ(*D_i_*)*cj*) are subsequently generated using the following formula:

(3) $$\Delta (D_{i})c_{j}=D_{i}-avg(D_{i})c_{j}$$

In this equation, $\Delta (D_{i})c_{j}$ is a deviation descriptor that actually measures to what extent a chemical structurally deviates from a set of compounds assigned as active and tested against the same experimental condition [11,81,82]. In the QSAR-Co software, the calculated *D_i_* descriptors are provided as inputs as these descriptors are automatically converted into $\Delta (D_{i})c_{j}$ by this tool for the development of mt-QSAR models [11]. 

### 3.3. Model Development and Validation

In the current work, we employed the QSAR-Co tool to develop mt-QSAR models by GA-LDA and RF methods [11]. Before setting up both these models, the dataset was divided into a modelling set and an external validation set by the *k*-means cluster analysis (*k*-MCA) technique [83] with the help of the STATISTICA software package [84]. The purpose of *k-MCA* is to ensure that the validation set covers the same chemical-biological space as the training set, on which the model is built with [60]. Constitutional descriptors calculated by AlvaDesc were used along with the *IAi*(*cj*) values for generating five clusters based on the Euclidian distances from 500 iterations. From each cluster, external validation set samples were randomly collected to form an external validation set of 160 data samples. It is worth mentioning here that, the mt-QSAR models were developed only with the modelling set of 566 samples. Once the models were developed with the modified descriptors as described in Equations (2) and (3), they were then used to screen the external validation set in order to estimate their true predictivity. The best predictive model was selected based on the predictivity obtained for the external validation set. For setting up the models, however, the modelling dataset was further randomly divided into a sub-training (80% of the training data) and a test set (20% of the training data) with the help of the QSAR-Co tool [11]. 

The parameter settings used for the GA-LDA technique in QSAR-Co were: (a) total number of iteration/generation: 100, (b) equation length: 10 (fixed), (c) mutation probability: 0.3, (d) initial number of equation generated: 100, (e) number of equation selected in each generation: 30. Although no data pre-treatment strategy was used during the development of the models, inter-collinearity of the model descriptors was checked by examining the cross-correlation matrix and models generated with highly correlated descriptors (−0.8 < *r* < 0.8, *r* being the Pearson’s correlation coefficient) were discarded. With the help of QSAR-Co [11] and STATISTICA [84], the internal predictivity of the models developed based on the sub-training set was evaluated by standard statistical indices such as the Wilks’ lambda (λ), chi-squared (χ^2^), the square of Mahalanobis distance (*D*^2^), Fisher’s statistic index (*F*) and the corresponding *p*-value (*p*)[85]. The goodness of prediction for the sub-training, test and external validation sets was evaluated by computing the following statistical measures: sensitivity (correct classification of the active cases), specificity (correct classification of inactive cases), accuracy (overall correct classification), *F*-measure and Matthews correlation coefficient (MCC) [57,85]. Moreover, a *Y*-randomization test was used on the sub-training set to judge the uniqueness of the statistical model [59,60]. Therefore, the values of the dependent variable were randomly scrambled 100 times, and the Wilk’s lambda (λ) of the original model was then compared with the average Wilk’s lambda (λ_rand_) of the randomized models. For determining the applicability domain, the standardization approach [61] was employed with the help of the QSAR-Co tool [11]. 

For settling the RF model, important parameters settings of QSAR-Co were considered, namely: (a) each bag size: 100, (b) maximum depth: 0 (unlimited), (c) number of randomly chosen features: 0 (i.e., n = int(log2[#Predictors]+1)), (d) number of iterations: 100. It should be noted that the change of these parameter settings failed to improve the predictivity of the modelling dataset to a considerable extent.

## 4. Conclusions

Multi-target QSAR modelling based on the Box–Jenkins approach was successfully utilised in recent years to establish a number of validated predictive chemometric models for various targets [9,10,11,50,60,82,86]. In the current work, we carried out such kind of mt-QSAR modelling on inhibitors of four different isoforms of class I PI3K enzyme using the recently introduced QSAR-Co tool [11]. Cell-based enzyme inhibition assay results obtained from chemical databases like CHEMBL are rarely utilised for the development of chemometric models. However, these results represent a more complicated bio-functional scenario that exists inside living cells, and therefore they may be more challenging to predict by using chemometric models. In the current investigation, our aim was to focus on the cell-based assays performed for PI3K enzyme inhibition. From one side, the developed mt-QSAR models help in understanding specific structural and physicochemical contributions responsible for higher potency. At the same time, the highly predictive models make them suitable for the screening of chemical libraries towards the search of novel hit molecules. The high statistical quality of the developed mt-QSAR-LDA model allowed us to calculate the relative contributions of some important cyclic/ring fragments towards higher PI3K inhibitory potential at various experimental conditions. Similarly, the model may also be employed to understand the contribution of aliphatic and other small structural fragments. The combination of the different in silico techniques employed in this work can serve as valuable guidelines to speed up early discovery of PI3K inhibitors. Finally, the current case study emphasises that the QSAR-Co tool may be utilised in the future to develop predictive models when response variables are dependent on multiple experimental conditions.

## Figures and Tables

**Table 1:** Overall performance of the final multitarget-quantitative structure–activity relationship (mt-QSAR) linear discriminant analysis (LDA) model.

Classification ^a^	Sub-Training Set	Test Set
ND_Total_	453	113
ND_active_	324	89
CCD_active_	310	84
Sensitivity (%)	95.68	94.38
ND_inactive_	129	24
CCD_inactive_	122	22
Specificity (%)	94.57	91.67
F-measure	0.967	0.960
Accuracy (%)	95.36	93.80
MCC	0.889	0.825

**^a^** ND_Total_—Number of total data-points; ND_active_—Number of active data-points, ND_inactive_—Number of inactive data-points CCD—Correctly classified data, MCC—Matthews correlation coefficient.

**Table 2:** Degree of collinearity among the variables of the mt-QSAR LDA model.

Descriptors	Δ[*nCONN*]*b_t_*	Δ[*Mor18m*]*b_t_*	Δ[*HTm*]*m_t_*	Δ[*nROCON*]*c_l_*	Δ[SpMAD_A]*b_t_*	Δ[*HATS2i*]*b_t_*	Δ[*HATS8s*]*c_l_*	Δ[*R1m*]*c_l_*	Δ[*F07*[*N-N*]]*c_l_*	Δ[*SM15_EA*(*dm*)]*c_l_*
**Δ** **[*nCONN*]*b_t_***	1.00	0.14	0.08	−0.20	−0.18	−0.29	−0.23	−0.17	0.00	0.07
**Δ** **[*Mor18m*]*b_t_***	0.14	1.00	−0.37	−0.18	0.15	0.11	0.21	0.14	−0.07	0.11
**Δ** **[*HTm*]*m_t_***	0.08	-0.37	1.00	0.09	−0.14	−0.29	−0.21	−0.07	0.14	0.13
***D*** **[*nROCON*]*c_l_***	−0.20	-0.18	0.09	1.00	0.01	0.17	0.00	0.02	0.23	0.12
***D*** **[SpMAD_A]*b_t_***	−0.18	0.15	−0.14	0.01	1.00	−0.06	−0.11	0.01	0.15	−0.26
***D*** **[*HATS2i*]*b_t_***	−0.29	0.11	−0.29	0.17	−0.06	1.00	0.18	0.02	−0.04	0.01
***D*** **[*HATS8s*]*c_l_***	−0.23	0.21	−0.21	0.00	−0.11	0.18	1.00	0.74	−0.09	0.29
***D*** **[*R1m*]*c_l_***	−0.17	0.14	−0.07	0.02	0.01	0.02	0.74	1.00	−0.02	0.36
***D*** **[*F07*[*N-N*]]*c_l_***	0.00	−0.07	0.14	0.23	0.15	−0.04	−0.09	−0.02	1.00	0.17
***D*** **[*SM15_EA*(*dm*)]*c_l_***	0.07	0.11	0.13	0.12	−0.26	0.01	0.29	0.36	0.17	1.00

Then, the Y-randomization test [59,60] was performed on the sub-training set to confirm that the model was not developed by chance. An average λ value of 0.978 was obtained from 100 randomized models in which the dependent parameter was randomly scrambled. As this average value is far greater than the original λ value (i.e., 0.234), one may conclude that the mt-QSAR-LDA model is unique in nature.

**Table 3:** Ten variables selected in the multi-target LDA model.

Name	Description	Descriptor Type
Δ[*nCONN*]*b_t_*	Number of urea (-thio) fragment, depending on the chemical structure and enzyme target	Functional group counts
Δ[*R1m*]*c_l_*	R autocorrelation of lag 1/weighted by mass, depending on the chemical structure and cell type	GETAWAY indices
Δ[*SpMAD_A*]*b_t_*	Spectral mean absolute deviation from the adjacency matrix, depending on the chemical structure and biological target enzyme	2D matrix-based adjacency matrix descriptors
Δ[*HTm*]*m_t_*	H total index/weighted by mass, depending on the cell mutation and chemical structure	GETAWAY H-indices
Δ[*F07*[*N–N*]]*c_l_*	Frequency of N-N at topological distance 7, depending on the chemical structure and cell type	2D Atom Pairs
Δ[*HATS8s*]*c_l_*	Leverage-weighted autocorrelation of lag 8/weighted by I-state, depending on the chemical structure and cell type	GETAWAY H-indices
Δ[*SM15_EA*(*dm*)]*c_l_*	Spectral moment of order 15 from edge adjacency matrix weighted by dipole moment, depending on the chemical structure and cell type	Edge adjacency indices
Δ[*HATS2i*]*b_t_*	Leverage-weighted autocorrelation of lag 2/weighted by ionization potential, depending on the chemical structure and biological target enzyme	GETAWAY H-indices
Δ[*nROCON*]*c_l_*	Number of (thio-) carbamates (aliphatic), depending on the chemical structure and cell type	Functional group counts
Δ[*Mor18m*]*b_t_*	Signal 18/weighted by mass, depending on the chemical structure and biological target enzyme	3D-MoRSE, weighted by mass

**Table 4:** Overall performance of the final mt-QSAR random forest (RF) model.

Classification ^a^	Sub-training Set (10-fold CV) ^b^	Test Set
ND_Total_	453	113
ND_active_	324	89
CCD_active_	313	87
Sensitivity (%)	96.6	97.75
ND_inactive_	129	24
CCD_inactive_	117	21
Specificity (%)	90.7	87.5
F-measure	0.965	0.972
Accuracy (%)	94.92	95.57
MCC	0.875	0.866

**^a^** ND_Total_—Number of total data-points; ND_active_—Number of active data-points, ND_inactive_—Number of inactive data-points, CCD—Correctly classified data, MCC—Matthews correlation coefficient. ^b^ 10-fold cross-validation statistics.

## Supplementary Materials

Supplementary materials can be found at https://www.mdpi.com/1422-0067/20/17/4191/s1.
